# Learning curves and outcomes of robotic colorectal surgery: A single-surgeon experience within a structured dual-console training program

**DOI:** 10.1007/s11701-025-03139-x

**Published:** 2026-01-12

**Authors:** Zsolt Madarasz, Krysztof Nowakowski, Michael Leitz, Bogdan-Cornel Sturzu, Anas Baltamar, Kira Baginski, Annika Hoyer, Miljana Vladimirov, Jens Hoeppner, Fabian Nimczewski

**Affiliations:** 1 Department of Surgery, Medical School, Bielefeld University, University Medical Center OWL, Campus Hospital Lippe, 32756 Detmold, Germany; 2 Biostatistics and Medical Biometry, Medical School OWL, Bielefeld University, 33615 Bielefeld, Germany

**Keywords:** Robotic colorectal surgery, Learning curve, CUSUM, RA-CUSUM, Dual console, Training program, Oncological outcomes, Single surgeon

## Abstract

Robotic colorectal surgery has gained broad acceptance, but defining the learning curve and safety profile during structured implementation remains essential. This single-surgeon study aimed to analyze procedural proficiency and oncological outcomes during the introduction of robotic colorectal surgery within a dual-console training framework. All robotic colorectal resections performed between September 2021, and December 2024 were retrospectively analyzed. Procedures included robotic anterior resection (R-AR), robotic low anterior resection (R-LAR), and robotic right colectomy (R-RC). Cumulative sum (CUSUM) and risk-adjusted CUSUM (RA-CUSUM) analyses were used to evaluate operative performance and safety. Demographic, perioperative, and histopathological parameters were analyzed descriptively. A total of 102 procedures were performed by the surgeon as the primary console operator (R-AR = 46, R-LAR = 32, R-RC = 24). Mean operative times were 163 ± 44 min (R-AR), 228 ± 56 min (R-LAR), and 154 ± 25 min (R-RC), respectively. Distinct learning curve turning points were observed for two procedures, with proficiency reached after approximately 28 R-AR cases and 16 R-RC cases. In contrast, R-LAR did not show a clear turning point but demonstrated a prolonged plateau between cases 6 and 27. Conversion rate was 1%, major complications (≥ Clavien–Dindo IIIb) occurred in 4.9%, and there were no intraoperative adverse events. The R0 resection rate exceeded 97% (R-AR: 100%, R-LAR: 96.9%, R-RC: 95.8%), and mean lymph-node yield was 28.2 ± 13. CUSUM and RA-CUSUM curves confirmed stable performance and consistent oncological quality throughout the learning phase. Robotic colorectal surgery can be safely implemented in academic centers within a structured dual-console training environment. Procedural proficiency was achieved after 28 rectal resections and 16 right colectomies.

## Introduction

Colorectal cancer (CRC) remains one of the most prevalent malignancies worldwide and a leading cause of cancer-related mortality [1]. Minimally invasive surgery has become the standard of care for most colorectal resections, offering faster recovery, lower complication rates, and comparable oncological outcomes compared with open surgery [2, 3]. However, laparoscopic total mesorectal excision (TME) remains technically demanding in the deep pelvis, where limited instrument articulation and visualization can compromise precise mesorectal dissection and autonomic nerve preservation.

The introduction of robotic-assisted surgery (RAS) has helped to overcome many of these limitations. Over the past decade, RAS has been increasingly adopted for both rectal and colonic procedures, with mounting evidence supporting its safety, reproducibility, and oncological adequacy. Robotic colorectal resections yield outcomes comparable to laparoscopy, with lower conversion rates and shorter learning phases [4, 5]. For right-sided colon cancer, robotic right colectomy (R-RC) has gained broad acceptance as an alternative to laparoscopy, particularly for complete mesocolic excision (CME) and intracorporeal anastomosis [6, 7].

Evaluating the learning curve is essential to ensure the safe and effective implementation of robotic colorectal surgery programs. Previous studies [8–10] have reported that proficiency in robotic rectal surgery is typically achieved after approximately 30–50 cases, although the exact threshold may vary depending on case complexity and the surgeon’s prior exposure to minimally invasive procedures. In contrast, R-RC with CME tends to exhibit a shorter learning phase, with proficiency generally achieved after 20–30 cases [11]. Analytic approaches such as cumulative sum (CUSUM) and risk-adjusted CUSUM (RA-CUSUM) analyses [12] are widely used to quantify learning progress and identify proficiency thresholds and performance plateaus. The concept of a surgical learning curve reflects the progressive improvement in operative efficiency and patient safety as experience increases. Recent evidence [8, 13] indicates that perioperative morbidity remains low even during early learning phases when robotic colorectal surgery is implemented within structured institutional training and proctoring frameworks.

Prior experience in minimally invasive colorectal surgery substantially accelerates robotic skill acquisition, as laparoscopic and open skills transfer effectively to the robotic platform, reducing operative time and early complications [14, 15]. Structured training programs that integrate simulation, wet-lab or cadaveric sessions, bedside assistance, and proctored dual-console mentoring enhance both the safety and efficiency of robotic implementation, ensuring a standardized transition to console independence [16–18].

Despite these advances, most published analyses assess single procedures—primarily robotic TME—while few evaluate multi-procedure learning trajectories within a structured robotic training program. Moreover, the quantitative impact of dual-console exposure and structured mentorship to skill acquisition remains underexplored.

Unlike most prior studies focusing on single procedures, this work provides a multi-procedure, single-surgeon analysis covering both rectal and colonic robotic resections within one structured training pathway.

Therefore, the present single-surgeon study aims to characterize the learning curves for robotic anterior resection (R-AR), robotic low anterior resection (R-LAR) and R-RC performed between September 2021 and December 2024 at a university hospital equipped with a dual-console *da Vinci X* platform.

## Materials and methods

### Study design and setting

This retrospective single-surgeon study was conducted at the Department of Surgery, University Hospital OWL – Campus Lippe, Bielefeld University. Between September 2021 and December 2024, 102 consecutive elective robotic colorectal resections were performed by a single surgeon who had completed structured basic robotic training. All operations were performed using a *da Vinci X* surgical system (Intuitive Surgical, Sunnyvale, CA, USA) with a dual-console configuration, which has been in use at the department since June 2018.

The study was approved by the Ethics Committee of the Westfalen-Lippe Medical Association and the University of Münster (Ref. No. 2024-790-f-S).

### Surgeon training and experience

The operating surgeon is an experienced colorectal specialist with a broad background in advanced laparoscopic surgery. Prior to robotic training, the surgeon had more than five years of experience in laparoscopic colorectal surgery, performing approximately 30 laparoscopic colorectal resections per year, including anterior rectosigmoid resections, low anterior resections, and abdominoperineal resections. Before performing robotic procedures independently, the surgeon completed a comprehensive, stepwise robotic training curriculum, including the official online training program on the da Vinci X system according to the Intuitive Surgical curriculum, simulator-based console training at our institution, dry-lab and cadaveric (wet-lab) exercises, and bedside assistance during multiple robotic colorectal procedures. The pre-robotic training phase lasted approximately six months. The initial robotic colorectal procedures were performed under direct proctoring supervision. Dedicated robotic colorectal training began in August 2021, initially focusing on R-AR and R-LAR with TME. R-RC with CME and intracorporeal anastomosis was introduced in early 2023. At our institution, open right colectomy was a standard training procedure, whereas laparoscopic right colectomy was not routinely performed prior to robotic implementation.

### Inclusion criteria

Only procedures that were robotically initiated and completed by the operating surgeon as the primary console surgeon were included in the learning curve analysis.

**Fig. 1:**
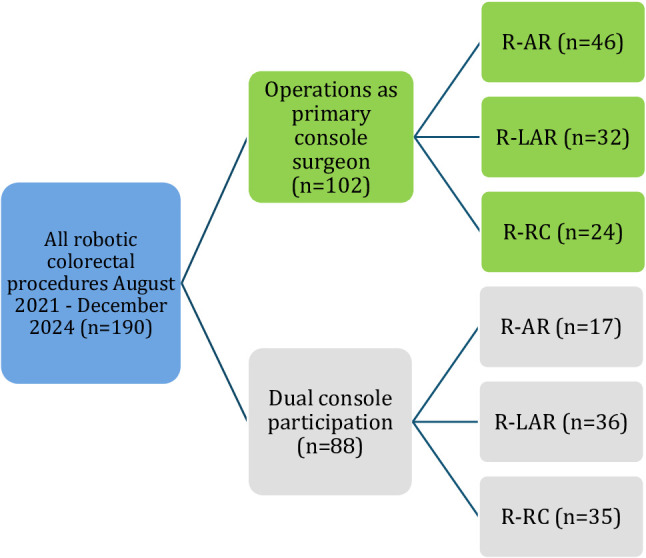
Flowchart robotic colorectal procedures, single surgeon

Eligible operations comprised R-AR, R-LAR, and R-RC, performed for colon cancer (right- or left-sided), rectal cancer, and sigmoid diverticulitis (Fig. 1).

### Patient management and operative standards

All patients with colorectal cancer were treated and staged in accordance with the current German S3 guideline for colorectal cancer. Treatment strategies were defined individually by a multidisciplinary tumor board comprising colorectal surgeons, gastroenterologists, oncologists, radiologists, and pathologists. For right- and left-sided colon cancers, preoperative endoscopic tattooing was routinely performed to facilitate intraoperative tumor localization. In patients with sigmoid diverticulitis, a preoperative colonoscopy was performed to exclude malignancy and assess the extent of disease. Mechanical bowel preparation and oral antibiotic prophylaxis were administered preoperatively according to institutional standards, and a single intravenous dose of prophylactic antibiotics was given at induction of general anesthesia. Intraoperative fluorescence angiography using indocyanine green (ICG) was routinely performed to assess anastomotic perfusion.

### Dual-console participation

In addition to primary console procedures, the surgeon also participated in dual-console operations during the same training period (Fig. 1). Dual-console participation involved both console observation and partial execution of predefined surgical steps, performed jointly with colleagues. These dual-console cases took place in parallel with the surgeon’s own primary console procedures and provided valuable opportunities for technical exchange, familiarization with specific procedural steps, and enhanced team coordination. Although these cases were not included in the CUSUM and RA-CUSUM analyses, they represented an essential component of the structured robotic training pathway, contributing to continuous skill development throughout the learning process.

### Study hypothesis

This study was designed to test the following hypotheses:

1. Each robotic procedure (R-AR, R-LAR, R-RC) demonstrates a distinct and identifiable learning plateau as experience accumulates.
2. A transfer effect occurs whereby prior experience in pelvic procedures (R-AR, R-LAR) shortens the learning curve for the subsequently introduced R-RC.
3. Exposure to dual-console training accelerates the development of surgical competency during the robotic learning phase.

## Variables and statistical analysis

Demographic, intraoperative, and postoperative data were collected for all patients.

Patient characteristics, perioperative parameters, postoperative results, and histopathological outcomes were analyzed descriptively. Continuous variables were reported as mean ± standard deviation (SD) or median (Q1; Q3), whereas categorical variables were expressed as absolute numbers and percentages. Pathologic evaluation included tumor (T), nodal (N), and metastasis (M) staging, tumor grading, R0 resection status, and assessment of specimen quality according to the Benz classification for CME [19] and the Quirke classification for TME [20]. Postoperative complications were graded using the Clavien–Dindo classification system [21].

To evaluate the progression of surgical performance, a CUSUM analysis was conducted using operative time (skin-to-skin, minutes) as the primary performance indicator. For each procedure, the deviation of operative time from the mean operative time of all cases performed with the same robotic procedure was calculated, and the cumulative sum of these deviations was plotted sequentially to generate individual learning curves for R-AR, R-LAR, and R-RC. The CUSUM score $\:{C}_{i}$ for the i-th operation [12]:

$$\:{C}_{i}={\sum_{i=1}^{n}}\left({X}_{i}-\overline{X}\right)$$

where $\:{X}_{i}$ represents the operative time of the i-th operation and $\:\overline{X}$ represents the mean operative time across all cases performed with the same resection procedure. The initial upward phase denotes the learning and adaptation stage, which is later followed by a turning point and subsequently a decline, indicating greater efficiency and superior operational performance. Because operative time may be influenced by patient and procedural complexity, a RA-CUSUM analysis was also performed. This analysis accounted for patient heterogeneity by estimating an expected operative time for each case using a multivariable linear regression model incorporating the following risk factors: Age (years), Body mass index (BMI, kg/m²), ASA physical status classification, and previous abdominal surgery.

The difference between the observed and expected operative times was cumulatively summed to generate a risk-adjusted learning curve for each procedure. This approach provided a more accurate assessment of performance progression while accounting for case mix and patient complexity. All statistical analyses were performed using R software (version 4.5.1).

## Results

### Patient characteristics

A total of 190 robotic colorectal procedures were performed during the study period, of which 102 were conducted by the surgeon as the primary console operator and 88 as dual-console participations (Fig. 1). Among the 102 primary console cases, 46 were R-AR, 32 R-LAR, and 24 R-RC (see Table 1). Overall, 59.8% of patients were male, and the mean age was 69 ± 11 years, with R-RC patients being the oldest subgroup (74.8 ± 6.8 years). The mean BMI was 26.6 ± 4.5 kg/m² and comparable across groups. Regarding indication, R-AR cases included both colon cancer (65.2%) and sigmoid diverticulitis (34.8%), while all R-LAR cases were performed for rectal cancer and all R-RC cases for colon cancer. The most frequent comorbidities were arterial hypertension (57.8%), type II diabetes mellitus (14.7%), and cardiac insufficiency (14.7%). R-RC patients showed the highest cardiovascular risk profile (25% coronary artery disease, 29% cardiac insufficiency, 46% anticoagulant use), representing a frailer subgroup.

**Table 1:** Baseline characteristics

	*R*-AR (*n* = 46)	*R*-LAR (*n* = 32)	*R*-RC (*n* = 24)	Overall (*n* = 102)
**Sex**				
Female	17 (37.0%)	11 (34.4%)	13 (54.2%)	41 (40.2%)
Male	29 (63.0%)	21 (65.6%)	11 (45.8%)	61 (59.8%)
**Age (Years)**				
Mean (SD)	67.2 (12.1)	67.2 (11.2)	74.8 (6.76)	69.0 (11.2)
**BMI (kg/m²)**				
Mean (SD)	27.2 (4.38)	25.8 (4.24)	26.5 (5.06)	26.6 (4.51)
**ASA Score**				
I	13 (28.3%)	0 (0%)	1 (4.2%)	14 (13.7%)
II	26 (56.5%)	15 (46.9%)	8 (33.3%)	49 (48.0%)
III	7 (15.2%)	15 (46.9%)	14 (58.3%)	36 (35.3%)
IV	0 (0%)	2 (6.3%)	1 (4.2%)	3 (2.9%)
**Indication**				
Colon cancer	30 (65.2%)	0 (0%)	24 (100%)	54 (52.9%)
Rectal cancer	0 (0%)	32 (100%)	0 (0%)	32 (31.4%)
Sigmoid diverticulitis	16 (34.8%)	0 (0%)	0 (0%)	16 (15.7%)
**Comorbidities**				
Typ II diabetes mellitus	7 (15.2%)	5 (15.6%)	3 (12.5%)	15 (14.7%)
Coronary artery disease	3 (6.5%)	2 (6.3%)	6 (25.0%)	11 (10.8%)
Cardiac insufficiency	6 (13.0%)	2 (6.3%)	7 (29.2%)	15 (14.7%)
Arterial hypertension	26 (56.5%)	14 (43.8%)	19 (79.2%)	59 (57.8%)
Renal insufficiency	1 (2.2%)	1 (3.1%)	2 (8.3%)	4 (3.9%)
Chronic pulmonal disease (COPD)	1 (2.2%)	1 (3.1%)	2 (8.3%)	4 (3.9%)
Smoking	11 (23.9%)	7 (21.9%)	5 (20.8%)	23 (22.5%)
Previous abdominal surgery	12 (26.1%)	4 (12.5%)	2 (8.3%)	18 (17.6%)
**Medical treatment**				
Anticoagulant treatment	9 (19.6%)	6 (18.8%)	11 (45.8%)	26 (25.5%)

### Operative outcomes

**Fig. 2:**
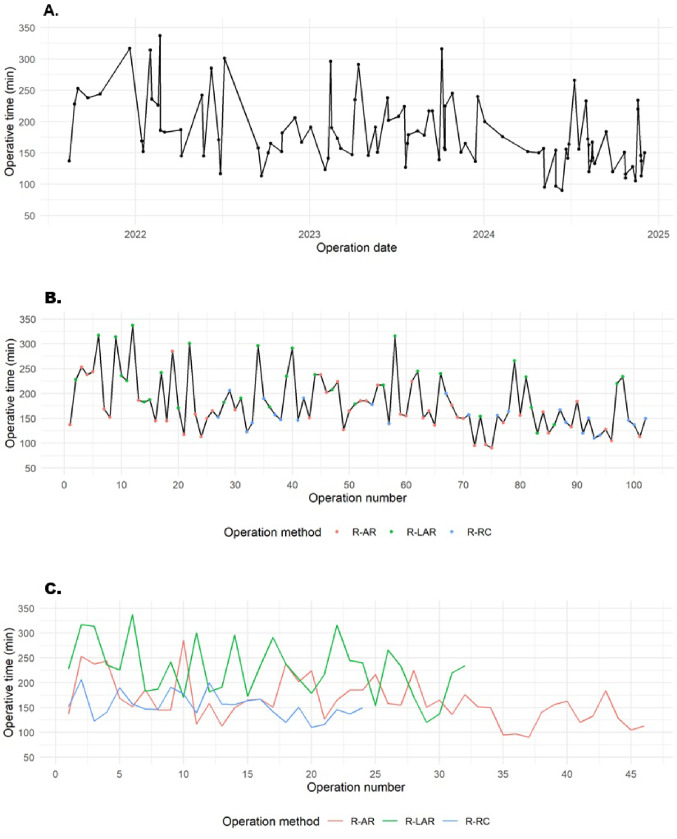
Operative times for all robotic colorectal procedures. (**A**) Procedural times between August 2021 and December 2024, shown chronologically by operation date. (**B**) Chronological duration of all procedures. **(C)** Procedure time by method, chronologically ordered

The mean operative time across all procedures was 181 ± 55 min, with a notable variation among the different subgroups: 163 ± 44 min for R-AR, 228 ± 56 min for R-LAR, and 154 ± 25 min for R-RC (Table 2; Fig. 2A-C). R-LAR required the longest operative times, reflecting the technical demands of deep pelvic dissection. The conversion rate was low (1%, one R-LAR case), and no intraoperative complications occurred in the R-AR or R-RC groups. Pfannenstiel incision was used for specimen extraction in nearly all patients (97.1%), demonstrating a standardized operative workflow.

**Table 2:** Procedural outcomes

	*R*-AR (*n* = 46)	*R*-LAR (*n* = 32)	*R*-RC (*n* = 24)	Overall (*n* = 102)
**Operative time (min)**				
Mean (SD)	163 (44.4)	228 (55.7)	154 (25.4)	181 (54.7)
Median [Q1, Q3]	156 [136, 185]	231 [183, 250]	151 [141, 165]	166 [145, 219]
**Intraoperative complications**	0 (0%)	2 (6.3%)	0 (0%)	2 (2.0%)
**Specimen extraction site**				
Pfannenstiel incision	46 (100%)	29 (90.6%)	24 (100%)	99 (97.1%)
**Conversion**	0 (0%)	1 (3.1%)	0 (0%)	1 (1.0%)

### Postoperative outcomes

Postoperative outcomes are presented in Table 3. Overall postoperative morbidity was 13.7% (Clavien–Dindo I–IIIa) and 4.9% (≥ IIIb). No anastomotic leakage occurred after R-AR or R-LAR, and one leak (4.2%) was observed following R-RC. The most frequent minor complication was postoperative bowel atony (9.8%), mainly in R-LAR (21.9%). Wound infection occurred in 3.9% of patients, and no postoperative bleeding requiring transfusion was observed. The reoperation rate was 3.9%, and the 30-day mortality was 1% (one R-LAR case). The mean hospital stay was 8.2 ± 3.0, longest after R-LAR (9.6 ± 3.3 days) and shortest after R-AR (7.0 ± 2.4 days).

**Table 3:** Post-operative outcomes

	*R*-AR (*n* = 46)	*R*-LAR (*n* = 32)	*R*-RC (*n* = 24)	Overall (*n* = 102)
**Post-operative complications**				
Anastomotic leak	0 (0%)	0 (0%)	1 (4.2%)	1 (1.0%)
Bowel atony	2 (4.3%)	7 (21.9%)	1 (4.2%)	10 (9.8%)
Chyle leakage	0 (0%)	0 (0%)	1 (4.2%)	1 (1.0%)
Pneumonia	0 (0%)	3 (9.4%)	0 (0%)	3 (2.9%)
Urinary tract infection	0 (0%)	0 (0%)	0 (0%)	0 (0%)
Wound infection	3 (6.5%)	0 (0%)	1 (4.2%)	4 (3.9%)
Reoperation	1 (2.2%)	1 (3.1%)	2 (8.3%)	4 (3.9%)
Clavien-Dindo I - IIIa	4 (8.7%)	8 (25.0%)	2 (8.3%)	14 (13.7%)
Clavien-Dindo ≥ IIIb	1 (2.2%)	2 (6.3%)	2 (8.3%)	5 (4.9%)
**Post-operative blood transfusion**	0 (0%)	0 (0%)	0 (0%)	0 (0%)
**30-day readmission**	1 (2.2%)	0 (0%)	1 (4.2%)	2 (2.0%)
**30-day reoperation**	2 (4.3%)	1 (3.1%)	0 (0%)	3 (2.9%)
**30-day mortality**	0 (0%)	1 (3.1%)	0 (0%)	1 (1.0%)
**Post-operative intensive ** **care unit (days)**				
Mean (SD)	0.261 (1.36)	0.656 (0.971)	0.333 (0.917)	0.402 (1.15)
Median [Q1, Q3]	0 [0, 0]	0 [0, 1.00]	0 [0, 0]	0 [0, 0]
**Post-operative intermediate ** **care unit (days)**				
Mean (SD)	0.304 (1.36)	0.344 (0.902)	0.0417 (0.204)	0.255 (1.05)
Median [Q1, Q3]	0 [0, 0]	0 [0, 0]	0 [0, 0]	0 [0, 0]
**Post-operative length of ** **hospital stay (days)**				
Mean (SD)	7.00 (2.39)	9.63 (3.29)	8.63 (2.76)	8.21 (2.99)
Median [Q1, Q3]	6.50 [5.00, 8.00]	9.00 [7.75, 11.0]	8.00 [7.00, 10.5]	7.00 [6.00, 9.00]

### Histopathological outcomes

The histopathological results refer exclusively to oncological cases and are presented in Table 4. R0 resection was achieved in 100% of R-AR, 96.9% of R-LAR, and 95.8% of R-RC cases; only two patients (2.3%) had R1 margins. The mean number of retrieved lymph nodes was 30.7 ± 15.6 for R-AR, 25.3 ± 11.8 for R-LAR, and 29.1 ± 9.81 for R-RC, with an overall mean of 28.2 ± 12.9. All specimens met the oncological quality standard of ≥ 12 lymph nodes. Positive nodal involvement (pN1-2) occurred in 33.3% of R-AR, 59.4% of R-LAR, and 29.1% of R-RC cases. Among all patients with colorectal cancer, UICC stages were distributed as follows: Stage I: 25.6%, Stage II: 29.1%, Stage III: 24.4%, and Stage IV: 20.9%.

**Table 4:** Histopathological outcomes in oncological cases

	*R*-AR (*n* = 30)	*R*-LAR (*n* = 32)	*R*-RC (*n* = 24)	Overall (*n* = 86)
**Type of the Tumor**				
Adenocarcinoma	30 (100%)	32 (100%)	24 (100%)	86 (100%)
**TNM pT-Stage**				
pT1	5 (16.7%)	3 (9.4%)	3 (12.5%)	11 (12.8%)
pT2	4 (13.3%)	4 (12.5%)	6 (25%)	14 (16.3%)
pT3	18 (60%)	21 (65.6%)	12 (50%)	51 (59.3%)
pT4	3 (10%)	4 (12.5%)	3 (12.5%)	10 (11.6%)
**TNM pN-Stage**				
pN0	20 (66.7%)	13 (40.6%)	17 (70.8%)	50 (58.1%)
pN1	6 (20%)	15 (46.9%)	2 (8.3%)	23 (26.7%)
pN2	4 (13.3%)	4 (12.5%)	5 (20.8%)	13 (15.1%)
**TNM pM-Stage**				
M0	24 (80%)	22 (68.8%)	22 (91.7%)	68 (79.1%)
M1	6 (20%)	10 (31.3%)	2 (8.3%)	18 (20.9%)
**Metastasis lung**	1 (3.3%)	3 (9.4%)	0 (0%)	4 (4.7%)
**Metastasis liver**	5 (16.7%)	8 (25%)	1 (4.2%)	14 (16.3%)
**Metastasis peritoneal**	1 (3.3%)	2 (6.3%)	1 (4.2%)	4 (4.7%)
**Resection status**				
R0	30 (100%)	31 (96.9%)	23 (95.8%)	84 (97.7%)
R1	0 (0%)	1 (3.1%)	1 (4.2%)	2 (2.3%)
**Tumor grading**				
G1	3 (10%)	3 (9.4%)	2 (8.3%)	8 (9.3%)
G2	26 (86.7%)	27 (84.4%)	20 (83.3%)	73 (84.9%)
G3	1 (3.3%)	2 (6.3%)	2 (8.3%)	5 (5.8%)
**UICC-Stage**				
1	7 (23.3%)	6 (18.8%)	9 (37.5%)	22 (25.6%)
2	11 (36.7%)	6 (18.7%)	8 (33.3%)	25 (29.1%)
3	6 (20%)	10 (31.3%)	5 (20.8%)	21 (24.4%)
4	6 (20%)	10 (31.3%)	2 (8.3%)	18 (20.9%)
**Quality of CME**				
0	0 (0%)	0 (0%)	24 (100%)	24 (27.9%)
1	0 (0%)	0 (0%)	0 (0%)	0 (0%)
Not analysed	30 (100%)	32 (100%)	0 (0%)	62 (72.1%)
**Quality of TME**				
1	0 (0%)	27 (84.4%)	0 (0%)	27 (31.4%)
2	0 (0%)	4 (12.5%)	0 (0%)	4 (4.7%)
3	0 (0%)	1 (3.1%)	0 (0%)	1 (1.2%)
Not analysed	30 (100%)	0 (0%)	24(100%)	54 (62.8%)
**Retrieved lymph nodes**				
Mean (SD)	30.7 (15.6)	25.3 (11.8)	29.1 (9.81)	28.2(12.9)

### Learning curves

**Fig. 3:**
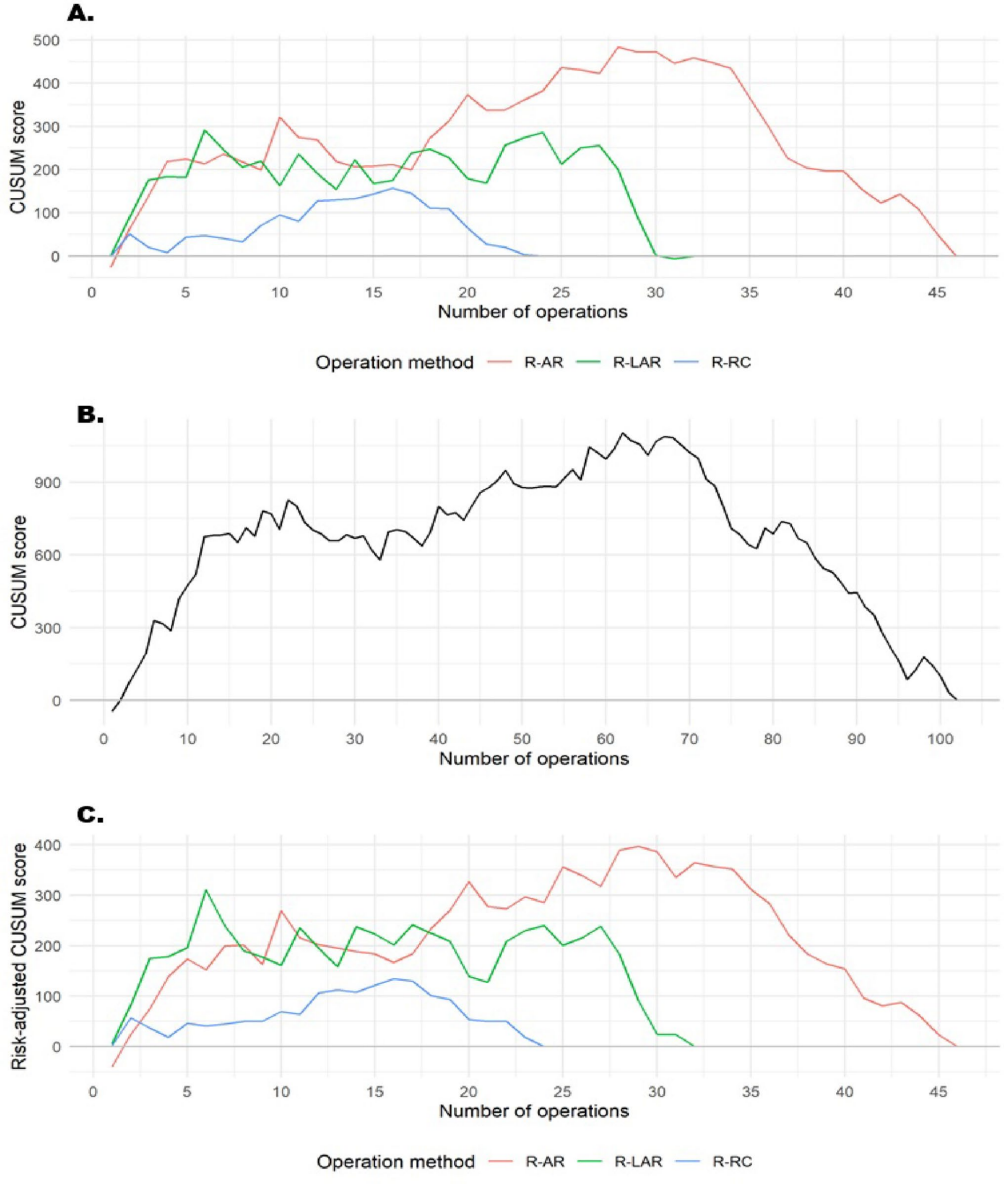
Procedure-specific CUSUM and RA-CUSUM learning curves.** (A)** CUSUM learning curves, categorized by operation method. (**B)** Overall CUSUM learning curve. (**C)** RA-CUSUM learning curve classified by operation method and the total number of operations conducted

Following the conversion of console time into CUSUM scores, the CUSUM learning curves for all three robotic resection procedures are represented in Fig. 3(A-C). The CUSUM curve associated with R-RC exhibited the slowest slope compared to other robot-assisted resection procedures, followed by R-LAR. The complexity associated with the cases, which may influence the duration of the operation, was similar across all procedures, as the RA-CUSUM curves reveal a pattern comparable to the CUSUM curves (see Fig. 3C).

R-AR represented the initial phase of the surgeon’s robotic colorectal training, beginning in September 2021. As shown in the CUSUM curve (Fig. 3A), the learning trajectory for R-AR displayed an initial upward slope during the first 28 operations, indicating the acquisition phase. Thereafter, the curve gradually declined, suggesting better operative performance and attainment of procedural proficiency. The RA-CUSUM curve (Fig. 3C)—adjusted for patient-specific risk factors—reveals a pattern that closely resembles the CUSUM curve.

R-LAR involved the most complex pelvic dissections, and as shown in Fig. 3A, the CUSUM curve displayed a short upward trend up to operation 6, which was subsequently succeeded by an extended plateau phase that continued until operation 27, followed by a decline, indicating the transition from the learning to the proficiency phase. The RA-CUSUM curve (Fig. 3C) showed an initial peak corresponding to these early cases, followed by a subsequent stabilization phase and a downward trend.

R-RC was introduced in early 2023, after the surgeon had already gained extensive robotic experience from rectal procedures. The CUSUM curve (Fig. 3A) demonstrated a slight initial rise and reached a turning point after approximately 16 cases, indicating a minimal additional learning requirement. The RA-CUSUM curve (Fig. 3C) shows a very similar pattern.

When all procedures were analyzed collectively, the overall CUSUM learning curve (Fig. 3B) showed an almost steady rise during the first 62 operations, followed by a progressive decline thereafter.

## Discussion

This single-surgeon analysis demonstrates that robotic colorectal surgery can be safely implemented within a structured dual-console training program, achieving high oncological quality and low morbidity even during the early learning phase.

CUSUM and RA-CUSUM analyses revealed distinct turning points for two of the three procedures: proficiency was reached after approximately 28 cases for robotic anterior resection (R-AR) and 16 cases for robotic right colectomy (R-RC), indicating rapid performance improvement and a clear transfer effect from prior rectal experience.

For robotic low anterior resection (R-LAR), no single distinct turning point could be identified. Instead, the learning curve demonstrated a prolonged plateau phase between approximately 6 and 27 cases. This pattern may be influenced by several factors, including narrow pelvic anatomy, tumor location, the need for splenic flexure mobilization, and the frequent use of neoadjuvant chemoradiotherapy, all of which are known to increase technical complexity and operative duration in rectal surgery.

### Comparison with previous literature

The learning curves identified in this study are consistent with previously reported thresholds for robotic colorectal surgery. In a single-surgeon analysis [8], proficiency was reached after approximately 44 cases, with stable outcomes and no increase in morbidity despite high-complexity procedures. Similarly, a multi-phase analysis [22] described three distinct phases—learning, competence, and mastery—with stabilization after approximately 80 cases, confirming progressive improvement in operative efficiency during transition from laparoscopy to robotics. A systematic review [5] comparing robotic and laparoscopic learning curves demonstrated that robotic approaches generally achieve proficiency after 15–55 cases. When comparing learning curves with previously published series, it is important to acknowledge heterogeneity in baseline laparoscopic experience, institutional case volume, and robotic training pathways. Differences in mentorship models and access to structured dual-console training may partially explain variations in reported proficiency thresholds across studies.

Our findings for R-AR (~ 28 cases) and the prolonged plateau observed for R-LAR (cases 6–27) are consistent with these reports but suggest a shorter adaptation period, likely reflects the benefits of a structured dual-console training environment and prior laparoscopic experience. For R-RC, a turning point was observed after only 16 cases, indicating a potential transfer effect from earlier rectal procedures. This aligns with CUSUM-based analysis [11, 23] which identified proficiency thresholds between 21 and 32 cases, confirming that right-sided procedures typically require shorter learning periods once robotic console familiarity is established.

Additional evidence [10] described a longer but safe learning curve for robotic proctectomy, requiring approximately 57 cases to reach operative times comparable to laparoscopic benchmarks. Nevertheless, perioperative morbidity remained stable throughout, supporting the conclusion that robotic colorectal surgery can be safely introduced within structured institutional programs. Collectively, these findings indicate that structured mentorship and prior minimally invasive experience substantially shorten the learning phase. Building on these observations, the present study further demonstrates that robotic colorectal surgery can be implemented safely with consistent oncological quality even during early training, as reflected by low complication rates and high R0 resection rates.

### Interpretation of outcomes

Despite being performed during the learning phase, perioperative and oncological outcomes in this study were comparable to established benchmarks reported from high-volume robotic centers [4, 24]. The conversion rate of 1%, the risk of major complications (≥ Clavien–Dindo IIIb) of 4.9%, and the absence of intraoperative adverse events confirm that robotic colorectal surgery can be implemented safely, even during early learning. These outcomes mirror those reported from large robotic TME programs [13, 25, 26]. The consistently high R0 resection rate (R-AR: 100%, R-LAR: 96.9%, R-RC: 95.8%) and mean lymph-node yield of 28 ± 13 exceeded oncological standards and were comparable with outcomes from larger institutional cohorts [24, 27]. These results confirm that robotic colorectal surgery can be safely introduced in academic centers through a comprehensive, supervised training program ensuring high oncological quality even during the early learning phase.

### Educational and technical aspects

The structured training pathway—including simulator-based training, wet-lab practice, bedside assistance, and dual-console mentoring—was decisive in accelerating competency acquisition. The dual-console system enabled real-time supervision and progressive autonomy, ensuring safe execution of critical steps. This experience corroborates earlier reports [16–18, 27] identifying dual-console systems as essential tools for effective robotic education and safe skill transfer.

### Limitations

This study has several limitations. First, its retrospective single-center design introduces potential selection bias and limits external generalizability. Second, the sample size—particularly within individual procedural subgroups—is relatively small and may affect the precision of estimated learning curve thresholds. Third, the study lacks a laparoscopic control group, which restricts direct comparison of robotic learning trajectories with those of conventional minimally invasive surgery. Finally, long-term oncological and functional outcomes were not assessed and should be evaluated in future prospective multicenter studies.

## Conclusions

Robotic colorectal surgery can be safely and efficiently implemented within a dual-console training framework. Procedural proficiency was achieved after approximately 28 rectal resections and 16 right colectomies, demonstrating a clear transfer effect from prior experience. Standardized curricula, simulation, and dual-console mentorship are likely essential components to ensure safety and accelerate skill acquisition in robotic colorectal surgery.

## Data Availability

No datasets were generated or analysed during the current study.
